# Application of PECARN rules would significantly decrease CT rates in a Dutch cohort of children with minor traumatic head injuries

**DOI:** 10.1007/s00431-020-03649-w

**Published:** 2020-04-28

**Authors:** Nicky Niele, Marlies van Houten, Ellen Tromp, J.B. van Goudoever, Frans B. Plötz

**Affiliations:** 1 Department of Paediatrics, Tergooi Hospital, Rijksstraatweg1, 1261 AN Blaricum, The Netherlands; 2 Department of Paediatrics, Amsterdam UMC, Emma Children’s Hospital,, University of Amsterdam, Amsterdam, The Netherlands; 3 Department of Paediatrics, Spaarne Gasthuis, Hoofddorp, The Netherlands; 4 Department of Epidemiology and Statistics, St Antonius Hospital, Nieuwegein, The Netherlands

**Keywords:** Computed tomography scan, Guidelines, Paediatric minor traumatic head injuries, Pediatric Emergency Care Applied Research Network

## Abstract

The aim of this study was to determine the potential impact of the Pediatric Emergency Care Applied Research Network (PECARN) rules on the CT rate in a large paediatric minor traumatic head injury (MTHI) cohort and compare this with current national Dutch guidelines. This was a planned sub-study of a prospective multicentre observational study that enrolled 1006 children younger than 18 years with MTHI. We calculated the number of recommended CT scans and described trauma-related CT scan abnormalities. The PECARN rules recommended a significantly lower percentage of CT scans in all age categories, namely 101/357 (28.3%) versus 164/357 (45.9%) (*p* < 0.001) in patients under 2 years of age and 148/623 (23.8%) versus 394/623 (63.2%) (*p* < 0,001) versus in patients 2 years and older.

*Conclusion*: The projected CT rate can significantly be reduced if the PECARN rules are applied. We therefore advocate that the PECARN guidelines are also implemented in The Netherlands.

**Table:** 

**What is Known:** *• To guide clinicians whether to perform a CT scan in children with a minor traumatic head injury (MTHI) clinical decision rules has been developed.* *• The overall CT scan rate in adherence with the Dutch MTHI guidelines is 44%.*	
**What is New:** *• The projected CT rate can significantly be reduced in a Dutch cohort of MTHI if the PECARN rules are applied.* *• The Dutch national guidelines for MTHI can safely be replaced by the PECARN rules.*

**Electronic supplementary material:**

The online version of this article (10.1007/s00431-020-03649-w) contains supplementary material, which is available to authorized users.

## Introduction

Several international clinical decision rules with high methodological quality have been developed to guide clinicians whether to perform or omit a CT scan in children with a minor traumatic head injury (MTHI), aiming to reduce the number of CT scans and thereby reducing the radiation risk [1–4]. External validations were performed in many settings and many countries to compare these rules for projected CT rates, diagnostic accuracy, and a cost-effectiveness analysis [5–7]. In The Netherlands, however, 2010 national guidelines are still used in clinical practice and strict adherence to these guidelines results in an overall high CT scan rate of 44% [8, 9]. In order to safely reduce this high number, it is methodologically more appropriate to determine the potential impact of one of these validated clinical decision rules on this CT rate [6]. Since the Pediatric Emergency Care Applied Research Network (PECARN) rules were designed to identify children at very low risk of clinically important traumatic brain injuries (ciTBI), we expect that this clinical decision rule will result in a significant potential reduction of the number of CT scans [1]. Hereby, we hope to reduce the amount of radiation and to decrease unnecessary management.

The aim of this study was therefore to determine the potential impact of the PECARN rules on the CT rate in a large paediatric MTHI cohort and compare this with current national Dutch guidelines. The outcome of our study may serve as a useful starting point for implementing these decision rules in The Netherlands.

## Methods

### Study design and patients

This was a planned sub-study of a prospective multicentre observational study that enrolled 1006 children younger than 18 years with MTHI who presented to six emergency departments in The Netherlands between 1 April 2015 and 31 December 2016 [9]. Exclusion criteria were incomplete data to compare guidelines on a case by case comparison.

### Guidelines

The Dutch national guidelines define several major and minor clinical criteria, specified by three age categories, namely under the age of two, between 2 and 5 years and 6 years or more (Supplemental Table 1) [1, 8, 9]. For children under the age of two, a CT scan was recommended if they had one or more major criteria. If a child under the age of two met one or more minor criteria, the options were a CT scan or clinical observation. For children aged between 2 and 5 years, the clinical decision rule was the same as for children under the age of two. For children aged 6 years or more, the presence of one or more major criteria or two or more minor criteria resulted in a CT scan. PECARN defines two age categories, namely under 2 years of age and 2 years and older [1]. A CT scan was recommended for children at high risk of ciTBI, while the advice for patients at intermediate risk was up to the clinician to decide whether to observe the patient or to perform a CT scan (Supplemental Table 1).

### Data analysis

We compared the number of recommended CT scans for both guidelines based on the presence of one or more major criteria according to the Dutch national guidelines and for patients at high risk according to the PECARN guidelines. The two age categories, between 2 and 5 years and 6 years and older, were combined for the Dutch guidelines. In case if the option was performing a CT scan or observation, we calculated the number of cases that fulfilled these criteria for both guidelines. All CT scans were interpreted by site radiologists.

### Statistical methods

For statistical analysis, we used SPSS Statistics for Windows, version 25.0 (IBM Corp, New York, USA). For comparing the clinical decision rules, a homogeneous group was created. This group included all children who presented within 24 h of head injury with GCS score > 13. The categorical variables between the groups were analysed using Pearson’s chi-square test or Fisher’s exact test when the expected frequencies were low. For all comparisons, an alpha value of < 0.05 was considered as significant.

## Results

Of 1006 eligible patients in our cohort, 26 patients were excluded due to incomplete data, and therefore 980 patients were included for a case by case comparison between the two guidelines.

### CT scan numbers indicated according to guidelines

#### Under 2 years of age

We found that based on the presence of one or more major criteria according to the Dutch national guidelines meant in 164/357 (45.9%) patients, a CT scan was indicated compared with 101/357 (28.3%) of patients with high-risk criteria according to the PECARN guidelines (*p* < 0.001) (Table 1). The number of cases that fulfilled the criteria to make a choice between a CT scan and observation was comparable between both guidelines (51.3% versus 52.9%) (Table 1).

**Table 1:** Amount of CT scan indicated by guidelines and actually obtained CT scans (adhered or non-adhered to the guidelines)

	< 2 years			≥ 2 years		
	PECARN (*N* = 357)	NATIONAL (*N* = 357)		PECARN (*N* = 623)	NATIONAL (*N* = 623)	
Indicated CT scans according to guidelines						
Indicated on high risk/major criteria	101/357 (28.3%)	164/357 (45.9%)	*p* < 0.001	148/623 (23.8%)	394/623 (63.2%)	*p* < 0.001
Indicated on intermediate risk/minor criteria^a^	189/357 (52.9%)	183/357 (51.3%)	NS	341/623 (54.7%)	96/623 (15.4%)	*p* < 0.001
Actual CT scans in adherence to guidelines (high risk/major criteria)						
CTs performed in line with guidelines	16/101 (15.8%)	35/164 (21.3%)	NS	74/148 (50.0%)	231/394 (58.6%)	NS
Abnormal CT scans	2/16 (12.5%)	7/35 (20.0%)	NS	10/74 (13.5%)	21/231 (9.1%)	NS
Actual CT scans in non-adherence to guidelines (no positive criteria or intermediate/minor criteria)						
CTs performed not in line with guidelines	26/256 (10.2%)	7/193 (3.6%)	*p* < 0.01	189/475 (39.8%)	32/229 (13.9%)	*p* < 0.001
Intermediate risk/minor criteria	24/26 (92.3%)	6/7 (85.7%)	NS	168/189 (88.9%)	31/32 (96.9%)	NS
No positive criteria	2/26 (7.7%)	1/7 (14.3%)		21/189 (11.1%)	1/32 (3.1%)	
Abnormal CT scans	5/26 (19.2%)	0/7 (0.0%)	NS	11/189 (5.8%)	0/32 (0.0%)	NS
Only applicable for PECARN: intermediate risk criteria	5/5	NA		11/11	NA	
Only applicable for PECARN: no positive criteria	0/5	NA		0/11	NA	

^a^Predictor variables leaving choice for the clinician for CT scan or observation

#### 2 years and older

Based on the presence of one or more major criteria or two or more minor criteria (additional criterion for age category 6 years or older) according to the Dutch national guidelines, CT scans were indicated in 394/623 (63.2%) of patients compared with 148/623 (23.8%) of patients according to the high-risk criteria of the PECARN guidelines (*p* < 0.001) (Table 1). The number of cases that fulfilled the criteria to choose between a CT scan and observation was significantly higher in the PECARN group (54.7% versus 15.4%) (*p* < 0.001) (Table 1).

### CT scan abnormalities

#### Under 2 years of age

The rate of CT abnormalities in adherence with the guidelines was not significantly different for the Dutch national guidelines versus the PECARN guidelines, namely 7/35 (20.0%) versus 2/16 (12.5%) (Table 1). In addition, non-adherence to the Dutch national guidelines resulted in no CT scan abnormalities. In the PECARN group, 26 patients had a CT scan not in line with the guidelines, of which 5 cases (19.2%) showed trauma-related abnormalities (Table 2).

**Table 2:** Clinical characteristics of children with trauma-related CT scan abnormalities

Case	Age	M/F	Trauma mechanism	Type of fall	Height of fall > 1 m	GCS	Dutch group (*n* = 980)	PECARN group (*n* = 980)	CT findings
							CT scan abnormalities (*n* = 28)	CT scan abnormalities (*n* = 28)	
							In adherence with Dutch guidelines	In adherence with PECARN guidelines	
1	< 2	F	Fall	From child carrier	Yes	15	Yes	Yes	ISF
2	< 2	M	Fall	From stairs	Yes	15	Yes	No	ISF
4	< 2	M	Fall	From child carrier	No	15	Yes	No	SF and ICH
5	< 2	F	Fall	From parent’s arms	Yes	15	Yes	No	SF and ICH
6	< 2	F	Fall	From stairs	Yes	15	Yes	Yes	SBF
7	< 2	M	Fall	From stairs	Yes	15	Yes	No	ICH
8	< 2	F	Fall	From bike seat	Yes	15	Yes	No	CON
9	> 2	M	Fall	From stairs	Yes	15	Yes	Yes	ISF
10	> 2	M	Fall	From parent’s arms	Yes	15	Yes	Yes	ISF
11	> 2	M	Fall	From stairs	Yes	15	Yes	Yes	ISF
12	> 2	M	Fall	On playground	Yes	15	Yes	Yes	ISF
13	> 2	M	Fall	From bench	No	15	Yes	No	ISF
14	> 2	F	Fall	From chair	Missing	15	Yes	No	ISF
15	> 2	M	Fall	From chair	Missing	15	Yes	No	ISF
16	> 2	F	Tripped with hopscotch			15	Yes	No	ISF
17	> 2	F	Fall	From gym equipment	No	15	Yes	No	ISF
18	> 2	M	MVC (scooter versus bus) with patient ejection			15	Yes	No	ISF
22	> 2	F	Fall	From horse	Yes	15	Yes	No	SF and ICH and CON
23	> 2	M	Fall from bike			14	Yes	Yes	SF and ICH
24	> 2	M	Fall	From bike seat	Missing	15	Yes	Yes	SF and ICH
27	> 2	F	Fall from bike			14	Yes	Yes	CON and ICH
28	> 2	F	Hockey ball to head			14	Yes	Yes	CON and ICH
29	> 2	F	Hockey ball to head			15	Yes	No	ICH
30	> 2	M	MVC (scooter) with patient ejection			15	Yes	No	ICH
32	> 2	M	Fall	From standing position	No (anticoagulant use)	15	Yes	Yes	ICH
33	> 2	F	Bicyclist struck by motorized vehicle (scooter)			15	Yes	No	SBF
34	> 2	M	Fall from bike		No	14	Yes	Yes	CON
35	> 2	M	Fall	From fence with free running	Yes	15	Yes	No	CON

*M* male, *F* female, *MVC* motor vehicle crash, *SF* skull fracture, *SBF* basilar skull fracture, *ICH* intracranial haemorrhage, *CON* intracranial contusion, *ISF* isolated skull fracture

#### 2 years and older

In adherence with the guidelines, trauma-related CT abnormalities were present on 21/231 (9.1%) CT scans in the Dutch national guidelines, compared with 10/74 (13.5%) in the PECARN guidelines, respectively. In addition, non-adherence to the Dutch national guidelines resulted in no CT scan abnormalities. In the PECARN group, 189 patients had a CT scan not in line with the guidelines, of which 11 cases (5.8%) showed traumatic-related abnormalities (Table 2).

## Discussion

In the present study, the recommended CT rate was significantly higher for both age groups in case the Dutch national guidelines were applied. The high number of CT scans can be explained by the greater amount of strict criteria to obtain a CT scan for the Dutch national guidelines compared with the PECARN rules. For example, isolated vomiting is a major criterion in the Dutch guidelines and consequently an indication to obtain a CT scan. However, traumatic-related CT scan abnormalities and ciTBI are uncommon in children who present with isolated vomiting after MTHI, and a management strategy of observation without immediate computed tomography appears to be appropriate [10, 11]. We choose for the PECARN rules since we expected that this would have the potential to decrease the number of CT scans. Others, who applied other clinical decision rules, observed the opposite. For example, Crowe et al. retrospectively applied the CHALICE rule outside the derivation sites at an Australian paediatric hospital and found that implementing this rule would double the number of CT scans [6].

In this study, we also calculated the number of cases in which the guidelines provided the option to choose between a CT scan and observation. In the children under 2 years of age, we found that a high percentage of children fulfilled these criteria according to both guidelines. In this age group, various studies report that clinicians prefer observation rather than a CT scan [12, 13]. For children 2 years and older, we observed that the number of cases was significantly higher according to the PECARN guidelines. Consequently, the absolute number of CT scans for children above 2 years of age according to the PECARN guidelines can fluctuate more according to clinician preferences and simultaneously give rise to more CT scans than in the Dutch national guidelines.

In our study, the CT rate of 44% in our cohort is significantly higher compared with other cohorts, which report a CT rate between 10 and 35% [1, 5]. Furthermore, in our cohort, CT scans were also obtained not in line with the guidelines. A remarkable finding in our study was when a CT scan was performed in cases where the criteria for a CT scan were not met, according to the Dutch guidelines, no trauma-related CT scan abnormalities were found in any of these CT scans. In contrast, if CT scans were performed not in line with the PECARN rules, we observed that 19.2% in children under 2 years of age and 5.8% in children 2 years and older showed traumatic-related abnormalities. However, we emphasize that the PECARN rules were not designed to detect trauma-related CT scan abnormalities but ciTBI.

Our study has several limitations. First, our sample size is too small to detect any ciTBI. The original PECARN study defines clinically important traumatic brain injury as death from TBI, neurosurgical intervention for TBI, intubation of more than 24 h for TBI or hospital admission of 2 nights or more for TBI, associated with TBI abnormalities on CT [1]. In the original cohort of 42.412 patients, the incidence of clinically important traumatic injury was 1.0%. Second, in the original PECARN cohort, patients were excluded in case of known brain tumours, pre-existing neurological disorder, neuroimaging at an outside hospital before transfer, ventricular shunt and bleeding disorder. In our cohort, none of all these exclusion criteria was applied. However, since these disorders have a very low incidence, we think that our cohort is not affected by these missing data and that our cohort still is representative.

The implications of our results for clinical practice in The Netherlands are in our opinion straightforward. We advocate that the current guidelines are replaced by the PECARN rules. We showed that the number of CT scans can significantly be reduced. Furthermore, many studies have already validated the PECARN rules on ciTBI in many countries and in many settings without safety concerns. Studies also demonstrated that the PECARN rules showed a very high sensitivity and specificity to detect ciTBI [5, 14–16]. The current decision rule with a low threshold to obtain a CT scan is not without risk. First, reported non-traumatic incidental findings on CT scans are high, up to 10%, which may pose medical and ethical considerations regarding management [18]. Second, there is a small risk of developing a radiation-induced malignancy later in life [19]. Third, it may also result in unnecessary management. For example, isolated skull fractures (ISFs) are the most commonly found abnormality on cranial CT scan in children with MTHI [17]. These children are at extremely low risk for emergency neurosurgery, intubation or death but are frequently hospitalized for a longer period [17]. The current evidence, however, strongly suggests no admission for all children with ISF following MTHI without clinical concerns [17].

## Conclusion

We found that the projected CT rate can significantly be reduced if the PECARN guidelines are applied. We therefore advocate that the PECARN rules are also implemented in The Netherlands.

## Electronic supplementary material

ESM 1 (DOCX 26 kb)

## Abbreviations

CATCH: The Canadian Assessment of Tomography for Childhood Head Injury rule
CHALICE: The Children’s Head Injury Algorithm for the Prediction of Important Clinical Events
ciTBI: Clinically important traumatic brain injury
CT: Computed tomography
GCS: Glasgow Coma Score
MTHI: Minor traumatic head injuries
PECARN: Pediatric Emergency Care Applied Research Network
TBI: Traumatic brain injuries
