# Supplementary material for: Application of PECARN rules would significantly decrease CT rates in a Dutch cohort of children with minor traumatic head injuries
Source: Eur J Pediatr. 2020 Apr 28;179(10):1597–602. doi: 10.1007/s00431-020-03649-w (PMC7479012; doi:10.1007/s00431-020-03649-w)
Supplement: Supplementary file 1 — (DOCX 26 kb) [file 431_2020_3649_MOESM1_ESM.docx]

**Supplemental Table 1. Inclusion and exclusion criteria, predictor variables and outcome measures of PECARN and Dutch NATIONAL guidelines**

|  |  | PECARN <2 years | PECARN ≥ 2 years | NATIONAL <2 years | NATIONAL 2-5 years | NATIONAL ≥ 6 years |  |
| --- | --- | --- | --- | --- | --- | --- | --- |
| Inclusion criteria |  | Age <18 y; presenting within 24 hours of head injury | Age <18 y; presenting within 24 hours of head injury | Age <18 y | Age <18 y | Age <18 y |  |
| Exclusion criteria |  | Penetrating trauma  GCS <14  *Known brain tumors*  *Pre-existing neurological disorder complicating assessment*  *Neuroimaging at an outside hospital before transfer*  *Patient with ventricular shunt*  *Patient with bleeding disorder*  *Trivial mechanism of injury* | Penetrating trauma  GCS <14  *Known brain tumors*  *Pre-existing neurological disorder complicating assessment*  *Neuroimaging at an outside hospital before transfer*  *Patient with ventricular shunt*  *Patient with bleeding disorder*  *Trivial mechanism of injury* | None | None | None |  |
| Predictor variables for CT scan | | **≥ 1 of the following high risk criteria** | **≥ 1 of the following high risk criteria** | **≥ 1 of the following major criteria** | **≥ 1 of the following major criteria** | **≥ 1 of the following major criteria** | **≥ 2 of the following minor criteria** |
|  | Mechanism of injury | None | None | High impact trauma of the head | High impact trauma of the head | High impact trauma of the head  Pedestrian/cyclist struck by vehicle  MVC with patient ejection | Fall from height |
|  | History | None | None | Posttraumatic seizure  ≥ 5 episodes of vomiting or vomiting for > 6 hours after trauma | Posttraumatic seizure  History of vomiting | Posttraumatic seizure  History of vomiting  Posttraumatic anterograde amnesia > 4 h after trauma  Anticoagulants use | Any LOC  Posttraumatic anterograde amnesia 2-4 h after trauma |
|  | Examination | GCS 14  Other signs of altered mental status^a^  Palpable skull fracture | GCS 14  Other signs of altered mental status^a,e^  Clinical signs of basilar skull fracture | GCS < 15  Suspicion basilar skull fracture  Focal neurological abnormalities  Scalp hematoma  Bulging anterior fontanelle | GCS < 15  Suspicion basilar skull fracture  Focal neurological abnormalities  Altered behavior | GCS < 15  Suspicion basilar skull fracture  Focal neurological abnormalities  Decrease of GCS with 2 points 1 h after presentation | External lesions of the skull  Decrease of GCS with 1 point 1 h after presentation |
| Predictor variables for CT scan or observation | | **≥ 1 of the following intermediate risk criteria** | **≥ 1 of the following intermediate risk criteria** | **≥ 1 of the following minor criteria** | **≥ 1 of the following minor criteria** |  |  |
|  | Mechanism of injury | Severe mechanism of injury^b^ | Severe mechanism of injury^b^ | Fall >1.0m or ≤1.0m with severe mechanism of injury  Fall on hard ground | Fall >1.0 or ≤1.0m with severe mechanism of injury | None | None |
|  | History | LOC for ≥ 5 s  *Not acting normally per parent report* | Any or suspected LOC  History of vomiting  Severe headache | Any LOC  <5 episodes of vomiting | Any LOC  Headache | None | None |
|  | Examination | Occipital, parietal, or temporal scalp hematoma (*non frontal*)^c^ |  | Altered behavior |  | None | None |
| Primary outcome |  | Clinically important TBI (ciTBI), defined as death from TBI, neurosurgical intervention for TBI, intubation of more than 24 h for TBI or hospital admission of 2 nights or more associated with TBI on CT^d^ | Clinically important TBI (ciTBI), defined as death from TBI, neurosurgical intervention for TBI, intubation of more than 24 h for TBI or hospital admission of 2 nights or more associated with TBI on CT^d^ | Neurosurgical intervention, transmission to a tertiary hospital or pediatric ICU | Neurosurgical intervention, transmission to a tertiary hospital or pediatric ICU | Neurosurgical intervention, transmission to a tertiary hospital or pediatric ICU |  |
| Secondary outcome |  | None | None | All abnormalities on CT scan^f^ | All abnormalities on CT scan^f^ | All abnormalities on CT scan^f^ |  |

*Data on critria in Italic text are not reported in the current cohort.*

*GCS,* Glasgow Coma Scale; *LOC*, loss of consciousness; *MVC,* motor vehicle crash

^a^Agitation, somnolence, repetitive questioning, slow response to verbal communication, ^b^Motor vehicle crash with patient ejection, death of another passenger, or rollover; pedestrian or bicyclist without helmet struck by motorized vehicle; falls >0.9m (or >1.5m for children **≥** 2 years of age); or head struck by high-impact object, ^c^Data on hematoma location were not present for all children, ^d^TBI on CT defined by any of the following descriptions: intracranial hemorrhage or contusion, cerebral oedema, traumatic infarction, diffuse axonal injury, shearing injury, sigmoid sinus thrombosis, midline shift of intracranial contents or signs of brain herniation, diastasis of the skull, pneumocephalus, or skull fracture depressed by at least the width of the table of the skull. Isolated non-depressed skull fractures were not assessed as TBI on CT, since they do not need specific therapy of hospital admissions [1], ^e^For children aged six years and older data were missing, since altered behavior was not registered for children aged 6 years and older in de current cohort, ^f^Abnormalities on CT scan excluded non-traumatic accidental findings. The following descriptions were defined as clinically relevant: intracranial hemorrhage or contusion, basilar skull fracture, complicated depressed skull fractures, cerebral edema or other relevant intracranial pathology. A linear isolated skull fracture was considered non relevant.
